# Humor in Times of COVID-19 in Spain: Viewing Coronavirus Through Memes Disseminated via WhatsApp

**DOI:** 10.3389/fpsyg.2021.611788

**Published:** 2021-04-01

**Authors:** Lucía-Pilar Cancelas-Ouviña

**Affiliations:** Departamento de Didáctica de la Lengua y la Literatura, Faculty of Education, University of Cádiz, Cádiz, Spain

**Keywords:** COVID-19, humor, memes, WhatsApp, cultural studies, digital cultural

## Abstract

The COVID-19 crisis, and its ensuing periods of confinement, has generated high levels of social stress on a global scale. In Spain, citizens were isolated in their homes and were not able to interact physically with family members, friends or co-workers. Different resources were employed to face this new stressful and unexpected situation (fitness, reading, painting, meditation, mindfulness, dancing, listening to music, playing instruments, cooking, etc.). Humor was one of the most frequent and widely used strategies in an attempt to keep perspective, deal with the seriousness of the situation and make the day-to-day more bearable. Humor is cultural: it varies from one country to the next and is part of the idiosyncrasies of a culture. It is deemed a particularly important feature of the Spanish personality. During the COVID-19 crisis, the main means or channels of communication were social networks. Throughout the confinement period, there was an excessive flow of humorous memes concerning Coronavirus and related shared experiences during the National State of Emergency decreed by the Spanish Government. The memes draw on irony, ingenuity and creativity to make a difficult and stressful experience more bearable. In this paper, a qualitative methodology based on ethnography research is used and ethnographic fieldwork is carried out on the memes disseminated through WhatsApp during the lockdown period experienced in Spain (14 March to 21 June 2020). The memes are considered to be an example of Netlore, digital contemporary folklore, and a theoretical framework on memes and humor is presented that discusses its different functions in order to channel grief, fear and suffering or to play down specific situations. A corpus of 644 memes that have flooded social networks are categorized and analyzed to witness how the Spanish have managed to bring out their humorous and creative side in difficult times even as they have criticized political decisions, let out their frustrations, described their new “normal” lives, interacted with others and anticipated the future.

## Introduction

On 14 March 2020, a state of emergency was declared in Spain due to the COVID-19 crisis. Surprisingly, and without warning, citizens faced an unexpected situation: a pandemic for which there was no precedent or instruction manual, and for which society lacked not only material, but also mental, resources. Coronavirus suddenly transformed the daily customs of a whole planet as citizens in many countries were banned from leaving their homes so as to avoid contagion and prevent worsening the pandemic (Romeo, 2020).

Spaniards are eminently social beings, renowned for their lively, friendly and outgoing character and their need for physical contact as they relate to their peers in day-to-day life. Spanish people spend much of their long day (they usually have dinner at 21.30 or 22.00) outdoors, interacting with others and enjoying the sun, favorable climate and natural surroundings. If the circumstances allow it, they are also fond of staying out late at night. The reader can imagine the distress Spaniards felt when on top of the stress of living through a pandemic, they were abruptly confined to homes that they were not accustomed to spending so much time in. Social networks became the only window to the world and they used them to relieve this lack of physical contact, an alternative way to keep in touch with their inner circle. Of all the gadgets and electronic devices, the mobile phone was crowned as the indispensable tool, and video calls and WhatsApps chats were the most popular communication channels during the confinement.

The Coronavirus pandemic created a gigantic network society (Castells, 2001), allowing individuals to develop their personal projects and aspirations by crossing over material limits of everyday life, both at home and work, thus creating networks of affinities beyond geographical limitations.

Social networks became the space where a common and familiar situation was shared to bring people together, and memes were the type of message most shared. This is not a trivial fact since one of the strategies most commonly used to play down the seriousness of a situation is the use of humor and, in this context, its purpose was to make lockdown more bearable. The memes became part of daily life and left behind a real-time chronicle of what took place. They showcased humor and creativity in difficult times. Bustos (2016) states that Spain is “a democracy dedicated to memes”^1^.

This paper explores a corpus of static online memes (we do not include videos, audios or gifs in the study), compiled in Spain, from 14 March to 22 June 2020, with regard to COVID-19. This subject matter is explored using a qualitative methodology and ethnographic fieldwork and the memes are categorized to reveal which fears, concerns and experiences regarding the Coronavirus crisis were addressed humorously. Romero de Vara (2020) says that it is possible to analyze a specific event through memes. In the last decade there has been a trend in research and studies in the fields of Sociology, Psychology, Education, Audiovisual Communication, Semiotics, Political and Cultural Studies, among others, centered on i-memes that support the type of study presented in this article.

The results obtained suggest that sending memes is about more than just entertainment, but rather a way of showing and channeling fears, sharing distressing experiences, socializing, and describing the “new normal” that Spaniards are facing from their own perspective through humor.

## Theoretical Framework

### Digital Memes: Structure and Characteristics

Etymologically the term meme derives from the Greek *Mimema* and means “what is imitated.” In its current meaning and usage it comes from English and was coined by ethologist and evolutionary biologist Richard Dawkins in 1976 in his book *The Selfish Gene* (Arango, 2015) to refer to a basic unit of cultural transmission that spreads easily among the population through speech, writing, behavior or any other phenomenon that can be imitated. This term is at the heart of a theory of cultural evolution: “memetics.”

Based on this general definition, the term internet meme or “i-meme” (Romero de Vara, 2020) has become popular to refer to the specific cultural unit that comes to life through the internet. The very nature of the internet, which allows information to be shared on a global scale in seconds, has contributed to the vast worldwide dissemination that memes have achieved today. They are created and modified by any user and deal with any aspect, situation or character anywhere in the world (Checa y Olmos, 2020). It is important to remember that in the 21st century, the internet is the main generator of popular culture, a barometer of what is fashionable.

Memes are artifacts of digital culture that offer information about the culture that creates and distributes them through multimedia text messages, video, gifs, etc. on electronic media such as social networks, showing, in an ironic or satirical tone, situations or everyday events in the life of the group of people who share them.

The Royal Spanish Academy of Language (RAE) defines meme as “Image, video, or text, usually distorted for caricatured purposes, which is disseminated mainly through the Internet.”

According to Ballesteros (2016) the term meme is used to refer to a verb or audio-visual composition, of a digital nature, that is transmitted over the network, and susceptible to evolution as it is transmitted virtually. Normally, they are jokes, phrases, occurrences or funny comparisons that are laid out with little elaboration to be broadcast on different social networks. Their creation favors templatization (Sandoval, 2017), understood as the use of design templates available on websites or meme-generating Apps that facilitate their easy creation and justify the aesthetics and similar designs they present. Some of the most used Apps for memes are Meme Generator Free, Meme Producer, Meme Factory, Memes for WhatsApp, Meme Creator, Memasik, Mematic, Modern Meme Maker, Meme Generator Pro 2019. These Apps allow the user to create a meme in seconds from a predetermined bank of images to which text is inserted. These digital tools use programming routines called macros (short for macroinstruction) and therefore fixed image memes are also called image macros (García Huerta, 2014). We also find the term memeplex to refer to a set or collection of memes.

Memes can be considered part of Netlore^2^, a term coined by Brunvand (1998) to refer to contemporary folklore transmitted over the internet. Netlore uses today’s digital media, including the mobile phone, to exist and perpetuate itself (Cancelas-Ouviña, 2010b). We can consider memes as a typology within contemporary folklore together with urban legends, hoaxes, mantras, jokes and chain letters among others. Shifman (2014) supports this idea considering memes as “(post) modern folklore” (p. 15). There is a website “Know your meme: http://www.knowyourmeme.com,” that researches and documents i-memes and viral phenomena. Founded in December 2008, “Know Your Meme” is conducted by independent professional editorial and research staff with the help of community members, contributing to elevate these samples of new folklore to the status of Netlore. There are currently several accounts and groups on social networks such as Facebook, whose only interest is the publication of memes, such as @Knowyourmeme, @iMemeflixx, @memes…

The most common form of memes combines image^3^ (photo, illustration, and drawings) with text and/or emoticons although other variants exist. According to their composition and design they can be classified into three types: text memes, Text+image memes and visual memes (Ballesteros, 2016 and Illustration 1 in the Supplementary Materials).

As far as the format is concerned, there are simple memes formed by a single image or text or the so-called “drakeposting” which is the succession of several images forming a sequence (Illustration 2 in the Supplementary Materials).

Most memes use Impact typography in white or comic balloons over an image. It is also frequent to use emoticons to complement texts. Many of them make use of the same image base or template and modify the text while it is also common to manipulate or modify existing images.

There are many authors who have written on the characteristics of memes (Vélez Herrera, 2012; Coscia, 2013; Ballesteros, 2016; Alarcón, 2017; Ruiz Martínez, 2018; Romero de Vara, 2020) and this serves as a reference for this study. Here is a list of characterizing elements that have been established for memes:

(1) Anonymous author: we do not know who the creators of the memes were. We read and share them without knowing who created them.
(2) Brevity: A brief message is immediately and easily understandable by the audience (Alarcón, 2017).
(3) Imitation: one of the most defining elements of memes (Dawkins, 1976).
(4) Humor: use of humor as an essential element of the message being transmitted.
(5) Ephemeral character versus longevity: usually memes are created, distributed and deleted by individual users from their devices, falling into oblivion. On the other hand, on a collective level, they can endure on the internet and become icons, in this case the opposite phenomenon would occur, with longevity being a characterizing property (Shifman, 2014). As Coscia (2013) points out, there are memes that are rapidly becoming extinct and others that survive because of their association with other memes.
(6) Digitalization: the i-meme is a digital work. ICTs allow its digitalisation which facilitates its creation and distribution through the internet and social networks.
(7) Immediacy: an event occurs and memes begin to be distributed on social networks at the moment that it is happening.
(8) Variation, selection and retention: in addition to being shared, they are deliberately altered in the process: different users re-appropriate and modify them to suit their particular communicative needs (Ruiz Martínez, 2018).
(9) Current affairs: memes tend to focus on current and fashionable subjects.
(10) Everyday life: memes usually portray everyday events.
(11) Relevance: the contents must have meaning or certain relevance for a specific group or for society as a whole in order to capture their attention (Vélez Herrera, 2012).
(12) Viral phenomenon: faithful copies of the i-meme replicated and shared easily (Romero de Vara, 2020). Large-scale and fast replication.
(13) Intertextuality, defined as a relationship of co-presence between two or more texts, that is to say, as the effective presence of one text in another (Genette, 1989). In the case of memes there is the copresence of images and elements of popular culture that are easily recognizable.
(14) Juxtaposition of provocative, bizarre or unconventional images in the same meme (Ruiz Martínez, 2018).
(15) Democratization in the production and distribution of satirical occurrences. A priori, anyone can design memes (Ballesteros, 2016).
(16) Multilateralism: memes are distributed in all directions with a great capacity for interaction.
(17) Poor design: From a graphics point of view, memes do not pay much attention to aesthetics: typographies and image quality are not taken care of and spelling mistakes abound on many occasions (Carpio-Jiménez et al., 2020).

Knobel and Lankshear (2007) believe that the three characteristics that would ensure the success of a meme would be firstly humor, followed by intertextuality with references to popular culture and juxtapositions of shocking and surprising things as a third element. Davidson (2012) in turn considers that for a meme to be successful it has to be communicable and malleable which would guarantee its dissemination: firstly in a spatial sense (many people disseminate it), and also in a temporal sense (many people reappropriate it and are able to find new meanings and uses for it), so the meme would remain in time and not go out of fashion.

As far as users are concerned, a distinction can be made between people who create content that is disseminated on social networks (creators) and users who consume and share it (consumers and distributors). It should be stressed that memes are produced by a minority but are shared by a large mass. It is assumed that imitation is an essential feature in meme context although a phenomenon called “plagiarism effect” can also be observed between memes, in the sense that identical memes appear with the same message and idea, which have been designed differently by the creators or re-creators. Memes are deliberately altered, appropriated and adapted by users according to their communicative intentions. The receivers feel free to appropriate, reproduce and modify them freely due to the fact that imitation is one of the key features for memes (Illustration 3 in the Supplementary Materials):

There is a hybrid category of users: “the prosumers” who are those with a dual role: producer and consumer of content. (Islas-Carmona, 2008).

## Humor and Creativity in Times of COVID-19

Of all the elements that make up memes, humor is the most characteristic and the one that best defines it. It is the differentiating feature with respect to other messages with a similar design that are spread through social networks. Cassany (2019) highlights this character of i-memes when he states that the purpose of these messages is usually to joke about current events: parodies, ironic comments or humorous exaggerations.

Humor is a characteristic of the human being that manifests itself continuously in daily life; however, not everyone has the same sense of humor and find the same situations funny. As González (2010) states, humor is a phenomenon of complex analysis and almost impossible to define because, apart from the personal aspects, it also requires historical, cultural and linguistic knowledge of the society in which a certain act of humor is inscribed. Whilst this paper will not delve into terminological issues establishing definitions of humor, laughter, parody, irony or satire, broadly speaking, humor will be simplified as everything that is capable of provoking hilarity: making us smile or laugh openly.

Based on the four types of humor proposed by Checa y Olmos (2020): (1) White or word humor, (2) Black humor, (3) Nonsensical humor, and (4) Graphic humor, we will define only graphic humor as this type best fits the humor contained in the memes. Checa y Olmos (2020) define graphic humor as a means of expression in which through drawings – sometimes without the aid of words – one plays with ideas and satirical or ironic intentions.

It is also necessary in this study to consider the differentiation between local humor and universal humor. Local humor is linked to a certain spatial and socio-cultural context. The humorous material arises from anecdotes, situations, facts or sayings that have taken place in that context, and would not make sense outside that context. It requires a specific complicity between the members of the community referred to with that humor. Universal humor is a humor without limits or barriers; it is an “exportable” humor of permanent effectiveness, easily understood by a wider audience (Illustration 4 in the Supplementary Materials).

The term “contingent humor” should also be clarified. It emerges from the context determined by the media and is usually political or social, often based on anecdotes, situations, facts or sayings reported by the press. Ephemeral by nature, it is a flower of a day that cannot last in a repertoire due to its rapid loss of validity. It is therefore more or less improvised, and for this reason it is not usually very elaborate or ingenious. Instead, it has the virtue of serving as a more or less critical showcase for current events. It is usually, although not necessarily, local humor. The dual character of contingent and local limits its understanding and effectiveness to the time and place in which it occurs; in that context it is quite assured of success, but outside it does not work. According to this definition, memes clearly present a type of contingent humor.

González (2010) proposes five main functions of humor: aggressive, sexual, social, defensive and intellectual. Memes have, without a doubt, a social function as their objective is to establish relationships and links between people. Ziv (1984) qualifies these functions of humor and specifies them by establishing five functions that are in line with memes and the proposal presented in this research: (1) Escape valve for social taboos, (2) Social criticism, (3) Consolidation of belonging to a group, (4) Defense against fear and anxiety, and 5. Intellectual play.

Martin et al. (2003) propose the idea of styles of humor, these being understood as the way in which people use humor. The styles of humor that they propose are: affiliative, self-enhancing, aggressive and self-defeating. Of these four styles of humor the ones that fit into the focus of this paper are the affiliative and the self-enhancing. Affiliative Humor is related to telling jokes, making playful and well-intentioned jokes, with the desire to relate to others, entertain and facilitate relationships. It is related to extroversion, interpersonal attraction, self-esteem, satisfaction with relationships and, in general, with positive feelings and emotions (Mendiburo and Páez, 2011). Self-enhancing humor refers to a humorous view of the world, in which the desire to be surprised by life and to maintain a humorous perspective on things prevails. It would be the closest measure to humor as a way of coping, since it allows distance to be kept from the stimuli that stress or generate problems (Lefcourt et al., 1995). During these months Spanish people have distanced themselves from COVID-19 and have related with their equals through an affiliative and self-enhancing humor.

**FIGURE 1:**
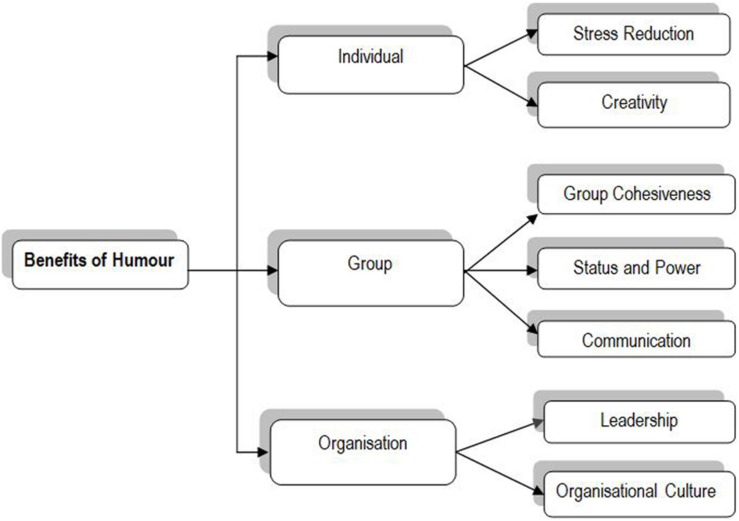
Benefits of humor based on Mathew and Vijayalakshmi (2017).

Although humor is a universal human experience, people of different societies perceive and use humor differently (Martin, 2007; Yue, 2010). There is nothing more characteristic of a people than the different manifestations of their sense of humor (Cancelas-Ouviña, 1997). Humor is a way of perceiving reality and depending on the culture we belong to, we will experience it in one way or another. Although it would be daring to classify humor in watertight compartments, creating national stereotypes about it, we must not forget that each country has a more or less generalized way of perceiving reality and of giving a common response to the things that make you laugh and amuse you (Cancelas-Ouviña, 2010a). For this reason, this research analyses how the Spanish society has perceived this health crisis from the perspective of humor (Figure 1).

Humor goes hand in hand with creativity. Mathew and Vijayalakshmi (2017) outline the benefits of humor at different levels: individual, group and organizational; highlighting creativity as one of the benefits of humor at the individual level and establishing a clear link between them.

According to Mathew and Vijayalakshmi (2017) there are numerous studies documenting the close relationship between humor and creativity. In turn, Carbelo and Jáuregui (2006) point out that humor stimulates creativity because it allows us to get away from problems, free the mind of unnecessary thoughts and emotions, and see things from new and unexpected points of view. A sense of humor brings out the funniest side of any situation, even a sad one, because it looks as it from an angle that makes us smile or laugh. It is essential to think creatively both to create and to perceive humor (Torres de Sánchez, 2009).

Memes show the inexhaustible creative ingenuity of society which, using humor in all its variables, (satire, irony, sarcasm, jokes, mockery, nonsense, caricatures, the absurd, puns, and parody) is capable of echoing ideas and feelings shared by society, of relativizing the conflicts, tensions and discomforts of everyday life and of distancing oneself from them. They also serve to criticize political decisions without bitterness or aggressiveness. This penetrates society quickly and attracts the attention of a broad social base. Every culture has its own sense of humor and Spanish humor is especially creative because it is witty, responsive and requires quick thinking.

The meme is a creative tool, as the creator gives free rein to his or her imagination, creative spark, and on many occasions plays with humor to make us aware of a fact, awaken our lateral thinking and bring us closer to reality from a different perspective.

There are certain features of humor that make a meme especially creative and clever. Taking into account that this research seeks to select creative samples to be included in the second part of this research, here is a list of humorous elements that contribute to a meme’s success:

- Unexpected relationships between situations and characters.
- Intertextuality.
- Inclusion of famous characters of the popular culture.
- Intelligent juxtaposition of images.
- Absurd hypotheses.
- Original metaphors and comparisons.
- Surprising facts.
- Play with double meanings of words.
- Use of language games: homophony.
- Hyperboles.
- Anachronisms.

Here is one specific example of a meme that can be analyzed to explain the creative process involved in meme design (Illustration 5 in the Supplementary Materials).

In this meme, intertextuality and the inclusion of famous people are used creatively. This sample exemplifies, in a funny way, the ravages that the passing of weeks in confinement wreaks on people. To do so, it plays with the physical resemblance of four famous people: the footballer Griezmann (1st Week) and the epidemiologist Fernando Simón (2nd week). The creativity lies in looking for images that reflect those physical resemblances that are recognizable to everyone but which show the ravages of time by presenting characters who are less and less physically favored (3rd Week: actor Marty Feldman and 4th Week: Actor Quique San Francisco) The creator plays with intertextuality and resorts to popular culture (football and film culture) to find the references with which to compare Fernando Simón, who is the main protagonist of the meme.

## Overview of the Research

Bañuelos and Pérez (2020) present three research approaches in relation to the study of memes:

(1) Studies that work on definitions, typologies and classification of memes.
(2) Specific studies that analyze memes in relation to humor, irony and their contents.
(3) Studies that analyze the behavior of memes according to their fundamental features and the effects of them going viral through social networks.

This research would be framed in the second line of investigation based on the works of Knobel and Lankshear (2007), Burguess (2008), Ballesteros (2016), Vesga (2016), Alarcón (2017).

This article presents a descriptive-interpretative study on the way the Spanish viewed Coronavirus using memes disseminated via WhatsApp. It follows a qualitative methodology with a digital ethnographic approach and socio-cultural perspective (Hine, 2004, 2005; Ardèvol and Gómez, 2012; Ballesteros, 2016; Romero de Vara, 2020). First, a case study (Yin, 1989) is carried out examining the behavior of those involved in the study when sharing and receiving memes. Subsequently, these digital artifacts are analyzed, focusing exclusively on online memes of a static nature.

The following research questions are studied with the aim of discovering, through these virtual popular folklore samples, the characteristic features of the memes produced in Spain during the pandemic:

(1) What do Spaniards laugh at and what makes them laugh in these difficult times?
(2) Which characters have the greatest presence in the COVID-19 memes in Spain?
(3) What elements of popular culture are most recurrent in these memes?

Selection criteria for corpus:

(1) Linked to the COVID-19 crisis.
(2) Geographical context: Spain.
(3) Humorous nature.
(4) Language: mainly Spanish or co-official languages (Catalan, Galician, Basque, or Euskera).
(5) If they are only visual memes, they must contain elements or characters from Spanish culture.
(6) Medium: whatsApp and Internet (Google).

For the selection of the corpus, the social network WhatsApp was selected because, although the memes are also widely spread by Facebook, Twitter or Instagram, it is the multimedia messaging application chosen most often by Spaniards^4^. WhatsApp messages are characterized by a rapid exchange of information capable of generating continuous data flows that are exchanged simultaneously in different chats, which makes it particularly suitable for this research (O’Hara et al., 2014).

This choice is also conditioned by the need to monitor the sample in a short time and to investigate an event at the same time that it is taking place. Furthermore, isolation during lockdown did not facilitate the use of other research instruments that required social contact such as interviews or focus groups.

Therefore, a methodological approach was selected to obtain our corpus from four sources and compile 644 memes regarding COVID-19 in Spain, providing enough material for analysis. The sample is classified into two large groups according to the way in which the samples were obtained:

Group 1: Memes obtained through interaction with the researcher^5^ in real contexts through the mobile phone, enabling the whole diffusion process to be observed in addition to the immediacy with respect to the events, the reception and reaction of the recipients and the capacity of the memes to go viral, since sometimes the same meme arrived simultaneously via several different groups.

Within this group we differentiate:

1.1. Memes from 17 different WhatsApp groups. There were 283 participants in these WhatsApp groups or chats and they share interests or have a common bond regardless of their degree of proximity or familiarity.
  The researcher has been interacting within these groups on a regular basis by capturing and collecting the samples over the past few months. These groups are very heterogeneous in terms of age, sex, political ideology, function of the group, professions, degree of kinship and relationship, belonging to different geographical areas in Spain, etc. There are two groups of university teachers, two groups of primary school teachers, two groups of parents of teenagers at different school ages, several groups with different relatives from different parts of Spain, a group with health personnel, several groups of friends, a group of leisure activities, a group of researchers, a group of social activists, a group of cultural gatherings, a mindfulness group, a religious group, a group of work colleagues.
1.2. Memes via WhatsApp received privately sent by friends, acquaintances and colleagues with whom the researcher maintains individual contact.

Group 2: Memes from the Internet:

2.1. Compilation of national online press memes to obtain a global vision of the phenomenon. This selection was conditioned by the editorial line of the different Spanish newspapers: national press (*El País, La Vanguardia, ABC, El Confidencial, El español*), sports press (*As, Marca, El Desmarque*), local press (*Diario Sur*). The online newspapers made a previous selection of the memes they included in their different articles.
  It is worthwhile to mention that *El País* newspaper, in its digital edition, has a section called “Verne” about the internet and social networks that dedicates a space to show and comment on the most popular memes of the moment <http://verne.elpais.com>.
2.2. Searches in google using the keywords “Covid-Spain” combined with different themes such as “masks,” “vaccines,” “overweight,” etc. provided a large volume of memes that came to swell the corpus obtained by the three previous sources.

A significant number of messages about COVID-19 have been excluded from this corpus because although they share the meme format, they were not of a humorous nature or were limited to: 1. Disseminate information on the disease and its prevention, 2. Recognize and appreciate the work of healthcare personnel and State Security Forces and Corps or highlight the good behavior of different social groups, 3. Show their opposition to the policies of the Spanish Government.

From the analysis of this corpus we have gone deeper into the humorous memes shared by a whole country during the first wave of the COVID-19 to draw conclusions. Despite the existence of all this humorous, funny and carefree material, this does not imply that Spanish society has been insensitive to the pain and harshness of the pandemic, nor has it been distant from the reality of the situation.

Timing of the study:

The search was limited to the period from 14 March 2020 to 21 June, when the state of emergency in Spain ended. This period is divided into three consecutive phases: (1) Confinement (14 March to 2 May), (2) Relaxing of restrictions (2 May to 21 June), 3. The new normal.

## Analysis of the COVID-19 Corpus Memes in Spain

The analysis of the corpus is split between the transmission process and the finished products:

(1) The transmission process: analysis of the behavior of WhatsApp users who share the memes during confinement in Spain. Case study on the behaviors of the research subjects.
(2) Analysis of the memes collected during this period.

Case study on the behaviors of the research subjects.

Firstly, this research focuses on the description and analysis of the human behavior observed in the 17 WhatsApp groups (283 people) that served as information sources, as the process of transmitting the memes is an element worthy of analysis in the COVID-19 crisis. The researchers did not report at any time that the research was being carried out so as not to interfere with the actions and interventions of the participants and the researchers limited themselves to interacting normally.

Observed behaviors will now be discussed. On the first day of confinement, March 13, there was a kind of collective hysteria on social networks. All sorts of messages were incessantly sent in an attempt to encourage, help, keep in touch, with memes taking a prominent place. This attitude is echoed by Checa y Olmos (2020) when they state that human language is different from other languages in that it is capable of combating hysteria with humor. In that first week, society was dependent on social networks as a palliative for the lack of physical contact, and memes undoubtedly became indispensable in the daily lives of many. Forwarding a meme is like telling a joke, sharing something that makes us laugh and that we think will also make the recipient laugh, and in the early days of lockdown, they were used to encourage others and in turn encourage the sender.

During the confinement, humor acted as a “Wellness Pill” (*píldora de bienestar)* (Ballesteros, 2016), as it was a brief stimulus that took the recipient out of the emotional impact of quarantine. It was a placebo for difficult moments. This would perhaps explain the excessive sending of memes in the first few days. Palacios (2020) states that humor is the candy to relax from this roller coaster of emotions. Memes worked, in Palacios’ words, as “sweetened pills” (*píldoras edulcoradas*) with the same effect as candy, and although humor cannot bring about a total change of mood, it can help you relax and disconnect from that emotional rollercoaster. Although reality did not change, it helped in the process of assimilation, releasing tension and dealing with uncertainty. In these early memes the Spanish people laughed, mainly, about their habits of mass shopping for supplies to cope with confinement and about what their new life was like. Let us not forget that laughter and smiles have a healing effect. Experts say that humor is a good therapy (Gardner, 1981; Porterfield, 1987; Martin and Dobbin, 1988), the brain releases endorphins and catecholamines, hormones associated with pleasure and happiness, it raises our spirits, helps us fight stress and strengthens the immune system, among other benefits (Urroz, 2005). As Sola-Morales says (2020) “humor through the Internet (…) emerges as an antithesis of fear, medicine for the soul, and as a political tool of struggle and social criticism” (p. 34).

It is noticeable to mention that the same meme (especially the scatological ones, or ones with religious element, or political bias) had an unequal effect among the members of different groups. In one group they produced a lot of laughter and were well received, and in others they produced rejection (either they were responded to with an emoji of rejection or there was no interaction with the meme).

Examples of religious memes that hurt the sensibility of some Catholics and were not well received in some chats are included below (Illustration 6 in the Supplementary Materials):

When the meme was good, it was transmitted quickly, reaching almost all the groups at once. In turn, it was common for the news to echo those fashionable memes that had spread throughout the country, making them newsworthy which in turn made them spread even faster (Romero de Vara, 2020).

Another interesting point is the selection made by the receiver prior to the reshipment. “Resend” is a subjective act that implies the recognition that something is funny and we believe that the person who will receive it will also find it comical, although sometimes this is mistaken, since the perception of humor is very personal and can sometimes be an object of conflict because it can even annoy others. People select content based on whether or not it fits their way of thinking or interpreting reality. When a meme is sent, it can be considered as a kind of quotation, the sender takes the meme as a subjective reflection of his or her worldview, which by forwarding it is shared by the sender. This selective process is easily observed when you belong to many WhatsApp groups simultaneously. Within the groups, different roles are also assumed: there are people who are more active and tend to be the ones who send the most memes (the Informants)^6^ while there are some very passive members who neither share memes nor give any feedback (Lurkers^7^ or Dippers^8^). Curiously, the most active members are those who are also the funniest, liveliest and most outgoing in personal relationships. In groups with similar political ideologies, certain memes were shared that would not be well received in other groups. The parents’ groups focused more on memes related to the attention and care of children in COVID-19 times. The same was true of the groups with some kind of work relationship. Each group filtered and shared those memes that had in common the point of union or purpose for which the group had been constituted.

April 7, 2020 marked a turning point in meme forwarding, as the company that controls WhatsApp took the decision in the midst of the COVID-19 pandemic to limit the forwarding of messages globally in order to reduce the overwhelming increase in forwarding at that time and to avoid hoaxes, fake news and messages that could contribute to misinformation. This decision caused a great deal of general unease in the country because in addition to being closed off, people perceived that they were now limited in their only means of expression at that time, social networks, and in particular, WhatsApp. There was a movement that proposed Telegram as an alternative channel to WhatsApp for communication, but it did not take hold and WhatsApp continues to have primacy. We were able to observe a drastic drop in the reception of memes and also how the general mood was negatively affected by this situation. Contacts were perceived to be increasingly sad, pessimistic and crestfallen, and they interacted less in chats. The groups were only reactivated when there was a change, a new extension, a phase in de-escalation, an unwise government instruction. However, as the messages had to be sent individually to each group and not collectively, the messages were more carefully selected and sent out, without a doubt, less.

This decrease in memes was gradual and less and less memes were received to the point where there are groups that are practically inactive since the new normal began and people started to go out and take up their lives again.

At the beginning of August 2020, the researcher asked the groups if they kept COVID-19 memes and if they could forward them. The answer was the same in all groups: they did not keep them. This served to corroborate the ephemeral nature of the memes. They are received and deleted. It does not make much sense to store images that occupy memory and that are not going to be used again. Perhaps the memes that reach our mobile phones do not last physically, but their effects and the memory do, since we sometimes comment on them as examples, since some of them become part of the collective imagination.

## Analysis of the Corpus of Memes Collected in Spain

This study included the following phases: collection (informants and search in networks), categorization into thematic typologies and analysis of the memes. The memes serve as a logbook of what was experienced over a specific period and a storytelling model was used to order them and select which events had the most impact in that specific period.

The memes were collected over 3 months by four sources as explained in the methodology section. Based on their analysis, an attempt has been made to characterize the humor shared by the Spanish population in relation to COVID-19.

The huge amount of memes has been classified to represent the topics observed. There may be cases where memes are in more than one category (Supplementary Table 1).

The main topics (Storytelling) found in the corpus on the Coronavirus crisis can now be analyzed to show how Spaniards have used humor in difficult times to express themselves.

COVID-19 in Spain had its own soundtrack, the song “*Resistiré*”*^9^* (I will resist) by the Dúo Dinámico, its own motto “Stay at home” and its symbol, a rainbow. On Friday 11 March the popular initiative *#QuedateenCasa* (StayAtHome) and *#FrenaLaCurva* (FlattenTheCurve) were launched and reinforced from the main media in the country. In the same vein, the Ministry of Health launched a crisis campaign with a series of contents in the media and also on digital platforms, trying to raise awareness through the hashtag *#EsteVirusLoParamosUnidos* (WeWillStopThisVirusTogether). Many memes have featured these emblems. The applause in windows and balconies at 8 p.m. was established on the second day of confinement and continued until the end of the whole process.

With regard to COVID-19 media personalities in Spain, undoubtedly, the epidemiologist Fernando Simón, director of the Spanish Coordination Centre for Health Alerts and Emergencies, is the face of Coronavirus in the country. Every day he was on television explaining the evolution of the virus. He gave the figures and, using a very didactic and approachable language, explained everything about the virus. Given the nature of some of his interventions and the examples used, his messages have been described as “simonadas” (Simonisms). His fame in Spain is widespread and he has featured in a large part of the memes on this subject. Rubio (2020) states that Simón has become, meme by meme, an icon of pop culture on the Internet^10^ (Illustration 7 in the Supplementary Materials).

The President of the Spanish Government, the Socialist Pedro Sánchez, has been another face of the crisis. He made lengthy speeches popularly known as “*Aló Presidente*” to explain the measures on television, which were the subject of many memes after each appearance. The memes offer a veiled criticism of the government’s management, the perceived lack of foresight and organization and the lack of effectiveness of the measures adopted (Illustration 8 in the Supplementary Materials).

The first few days, the Spanish population raided the supermarkets to stock up on food. One of the most surprising bulk purchases, and for which no explanation has yet been found, was toilet paper, and this also featured heavily in the memes (Illustration 9 in the Supplementary Materials).

Another recurrent theme was the putting on of weight, resulting from lack of exercise and excessive food intake. Most Spaniards gained weight during the confinement either due to eating out of boredom or anxiety, because they had more time to cook, or, if the lack of flour and yeast in the supermarkets was anything to go by, because they suddenly became fond of baking (Illustration 10 in the Supplementary Materials).

Another issue that worried Spanish citizens was the lack of sanitary material (tests, masks, protective material). A batch of COVID-19 tests was purchased from China which turned out to be defective. This was another source of funny jokes, hoaxes, pranks and tricks on social networks. The lack of PPE (Personal Protective Equipment) for health workers was the subject of many memes. They focus, with irony, on the lack of confidence the population had in the ability of the government to provide the necessary equipment for health workers. It is necessary to explain that the acronym for PPE in Spanish is EPI (*Equipo de protección individualizada*) and that the name for the character of Sesame Street, Bert, in Spanish is EPI too (Illustration 11 in the Supplementary Materials).

Spanish citizens are social beings, they spend a lot of time in the street and living in confinement was a challenge. Many memes made jokes about the picaresque used in order to go for walks: one of the main excuses was to walk the dog as this was allowed by the government (Illustration 12 in the Supplementary Materials).

Working from home, video calls even to the doctor and online teaching all became the norm and memes reflected that reality. They provided many laughs as citizens shared a common reality, even though it was also often a source of much stress and anxiety (Illustration 13 in the Supplementary Materials).

Popular festivals and traditions are strongly rooted in Spanish culture and citizens enjoy participating in them. COVID-19 caused many of these festivals, such as Holy Week (*Semana Santa*), the *Fallas* of Valencia, the *Sanfermines*, the *Feria de Abril*, and *El Rocío* to be canceled and there were memes to reflect this. The suppression of these festivals had a great impact socially and, for many Spaniards, it was a source of great sadness. To alleviate the situation, the government, when announcing the suspension, spoke in principle of postponing these festivals until autumn, not of canceling them, although in the end they were never held. This particular group is made up of images provided by the creators (photos or illustrations) and they do not come from the image banks of the Apps to create memes since they are very local and specific events (Illustration 14 in the Supplementary Materials).

Taking care of children during COVID-19, reconciling family and work life and homeschooling have been others topics including in the corpus. In some cases, grandparents had to help many families and the memes satirized the situation of them as babysitters (Illustration 15 in the Supplementary Materials).

The mask became an indispensable part of our outfit and many memes have been dedicated to it. In the beginning, getting a mask was a risky sport and many memes portrayed the ingenious solutions to alleviate the shortage. On returning to the new normal, we had to pay attention to our physical appearance and all the aesthetic issues we had neglected such as hair care, eyebrows and hair removal. The new normal worried us and the social distancing, masks, hand washing with hydroalcoholic gels, gloves and the vaccine now became the main meme protagonists (Illustration 16 in the Supplementary Materials).

## Discussion

In the corpus analyzed, the characteristics included in the theoretical framework of this study are present: humor, intertextuality, juxtaposition, templatization, current events and everyday life, and the viral nature. Humor is the predominant element. According to Romero de Vara (2020), sarcasm, humor and parody have got a huge potential to convict, influence and condition the decisions of those who are exposed to them. It is worth emphasizing that the meme creators in this context have known how to describe reality with ingenuity.

In light of the data collected, we can conclude that the category most represented in the sample deals with the political leaders that have managed the crisis. 11.52% of the memes were attributed to this category, followed by the virus and its effects (9.68%). Life in lockdown and purchases and supplies is also a prominent group (8.60%).

According to social networks, the celebrity of Coronavirus in Spain was undoubtedly Fernando Simón, who became a popular culture icon with a legion of followers on social networks, followed by the Prime Minister Pedro Sánchez and to a lesser extent the Minister of Health, Salvador Illa. The Vice President of the Government, Pablo Iglesias and the Minister of Equality, Irene Montero were also the subject of several memes.

Local customs, festivals and traditions have been reflected in the memes, assuming with some regret that in 2020 they would not be celebrated. There is a group of memes about Seville’s Holy Week that are only relevant to a very specific population.

Memes encourage forms of intertextuality by combining elements of popular culture and references to politics to emphasize the dynamics of the real and virtual world (Shifman, 2014). They are strongly linked to popular culture (Brown, 2009) or, more specifically, to mass culture (Carroll, 2002). There are icons and international characters from popular culture such as the Simpsons, Julio Iglesias, Rambo, Will Smiths, Darth Vader, Sean Penn, Bert (Sesame Street), the Girl from the Exorcist, Masada, Queen Elizabeth II of the United Kingdom, Pope Francisco, Mr. Bean, Batman, Sheldon Cooper, Marty Feldman, Michael Jackson, Superman, Barbie dolls, Maranon, the Joker, Antoine Riemann, Gollum, John Travolta, Arnold Schwarzenegger, Marty and Brown from Back to the Future which appear recurrently in these COVID-19 memes.

There is also a large group of memes with a transtexual character that use pictorial works creatively to make comparisons and humor. Paintings by Diego Velázquez, Francisco de Goya, Fernando Botero, Vicent Van Gogh, Leonardo Da Vinci or sculptures by Michelangelo or the Vigeland Park have served to parody the crisis of COVID-19. This use of art in memes is especially creative and there is a group of memes in this line worthy of future analysis.

Although there are some memes that, if translated into other languages, could be extrapolated since COVID-19 is a worldwide pandemic, 75% are very local because they feature Spanish characters, satirize situations experienced in Spain, criticize actions of the Spanish government, reflect typical Spanish habits and behaviors, or make comparisons with advertisements, television programs, songs or characters from Spanish popular culture. This would make them difficult to understand for people of other nationalities. Likewise, there are memes starring Spanish TV presenters, Iker Jiménez, Pedro Piqueras and Jordi Hurtado, members of the Royal Family including Queen Letizia and Queen Sofía, the humorous character “la vieja del Visillo” or Torrente, the president of Real Madrid, C.F Florentino Pérez, the bullfighter Curro Romero, the motorbike rider Mark Márquez, the former Spanish dictator Francisco Franco, the pop singer Sergio Dalma, the actor Quique San Francisco, and comic characters Mortadelo and Filemón. There is an allusion to the boy with the drum from Spain’s Got Talent^11^, to Cofidis, to the “in-love” girl in the advertisement for Pizza Casa Tarradellas or to supermarkets such as Mercadona or shopping centers such as El Corte Inglés. For humor to be given and perceived, it is essential that the receiver masters all the codes and has all the information so that it is relevant and the irony, satire, wordplay, metaphors, comparisons, or witty ideas can be perceived and understood, in the hope to provoke a smile.

But the main protagonist of these humorous memes is “the us in confinement,” the common people, the community that is living this experience and that seeks ways to escape in order to de-dramatize and relativize the seriousness of the situation and see it from a distance by presenting a known, familiar and easily recognizable reality in a different light.

The way in which the government has managed the health crisis has provoked an inexhaustible source of memes, especially with regards to the lack of medical equipment and the relaxing of restrictions. The meme has been used as a tool to show social weariness regarding the problems faced and the discontent of the Spanish people who parodied everything they were experiencing. The population in general, and young people in particular, assert their opinion with satire and irony through the memes. A great deal of attention is given to the perception of unwise management by the government, the continuous changes of opinion by leaders and a perceived lack of means, resources and foresight to face the crisis. Despite this, the population is generally thought to have patiently abided by all the rules, only expressing its discontent through social networks.

A certain amount of animosity towards the people of Madrid, who are the subject of many memes, has been detected in the memes. Madrid was one of the major centers of the disease during the first wave of COVID-19, and the inhabitants of other towns and cities objected to the idea that people from Madrid should be able to move around the country and thus contribute to spreading the virus.

“Portmanteau”^12^ words for new situations have been observed such as neologisms like “covidiota,” “Balconazi” “confitamiento” o “cuarenpena”^13^ are reflected in some memes.

In the 644 memes corpus, there are only two that are tangentially linked to football. This is surprising as this popular sport is usually the subject of many memes in the country but went almost unnoticed during the pandemic. This is perhaps due to the suppression of the Spanish Football League and the Champions League which meant that football was not in people’s minds during those months.

When the meaning of a meme is understood, it can meet some of our greatest human psychological needs by making us feel safe and giving us a sense of belonging to the community (Malo et al., 2010). Affiliative and self-enhancing humor, fostered by memes, have a cohesive element and during confinement they were used them to create the feeling of being accompanied as a way of coping with the isolation and physical distancing imposed. The meme was very useful, socially speaking, because it was a form of catharsis that helped release pressure and tension felt, and sometimes served to channel anger and rage in the face of the sense of powerlessness produced psychologically in a highly complex situation.

## Conclusion

This paper has presented a sample of Spanish humor, a differentiating feature of the national personality, reflecting the issues laughed at during the COVID-19 health crisis in order to keep up spirits. The great Spanish creative vein is displayed in the infinity of memes that draw on occurrences and events experienced with humor. As Lynch (2002) states “jokes and humor play an important role in determining who we are and how we think about ourselves, and how we interact with others” (p. 425).

The humor of the memes collected in this research can be defined as local, graphic and contingent with specifically social, affiliative and self-enhancing functions.

We affirm that i-meme is a typology of netlore and that it can be considered a manifestation of contemporary folklore. It fulfills many of the characteristics of folklore (anonymous, popular character, variants compatible with the transmission process coexist, collective character…) with the only difference being that transmission is carried out through the internet and social networks.

This exhaustive analysis of a voluminous corpus demonstrates, through social networks, that even when Spain panicked, it did not lose its sense of humor. Taking things as they come with humor, even if they are not to our liking, is a privilege for us as intelligent beings. A multitude of situations, opinions and emotions have been described, reflecting what was newsworthy and going viral within a few hours. It is worthy to note that no black humor about death was encountered in the memes, nor did they refer to the nature of the victims, the sick or the cruelty of the illness. Rather than refer to these areas, the Spanish focused on revealing features of their new daily life.

As Hall (1997) states, the meme is capable of conveying concepts, ideas or feelings and therefore is able to construct meaning and communicate significance. During the COVID-19 crisis, the Spanish communicated small things that frightened them, made them feel uncomfortable, things they missed, that made them angry or caused them anxiety, and they tried to diminish the seriousness of the situation with a smile. During those months it was often stated that “I laugh so I don’t cry.” On many occasions, laughter was directed at some absurd behavior or at how ridiculous concerns of yesteryear seemed. Humor in COVID-19 times had a cathartic, liberating, soothing and above all healing effect. It became a psychological lifesaver in the hardest and most difficult moments and the creative vein was exploited to reveal the new normal through memes.

Future research could compile a systematic review of articles published on this theme (Abidin, 2020; Carpio-Jiménez et al., 2020; Flecha et al., 2020; Hussein and Aljamili, 2020; Kertcher and Turin, 2020; Sola-Morales, 2020; Norstrom and Sarna, 2021), analyzing how COVID-19 is viewed through memes in different countries to see if there are some common aspects in the memes circulating worldwide and to highlight the differentiating elements by identifying features that cause the same reality to be perceived differently.

## Data Availability Statement

The raw data supporting the conclusions of this article will be made available by the authors, without undue reservation.

## Author Contributions

The author confirms has being the sole contributor of this work and has approved it for publication.

## Conflict of Interest

The author declares that the research was conducted in the absence of any commercial or financial relationships that could be construed as a potential conflict of interest.

## Footnotes

1 In http://www.elmundo.es/opinion/2016/01/22/56a129a1268e3ed2018b4575.html

2 Le Quellec and Motlow (1997) uses the term “Screenlore” instead of netlore to refer to a modern popular folklore mediated by the cultural industries through the screens of communication and information technologies.

3 All the illustrations included in this work are indispensable since they constitute the central axis of the study. Given the anonymous nature of memes that are transmitted virally through the networks, it is impossible to know the authorship of them and therefore to request any permission for their publication. We can attest that the memes collected for this research have not been altered or modified by faithfully reproducing the samples collected.

4 According to the *Centro de Investigaciones Sociológicas* (CIS) Barometer of July 2020, 91.8 percent of Spaniards have a mobile phone and according to the Barometer of 2016, 98.1 percent of users choose WhatsApp for multimedia messaging.

5 No individual informed consent has been sought from study participants because it was vital that they were unaware that this research was being conducted so that they would not intentionally contaminate and alter the process of Netlore transmission. However, we emphasize that no personal information or data has been requested that would infringe on their privacy. We simply collected the memes that were sent to the group, focusing on the meme and not on any of the subjects, always protecting their anonymity. We have respected the Data Protection Act at all times. After finishing the research period (March-June) we informed them in the chats offering to show the results of the study. This research follows the ethical code of the University of Cádiz (Spain) (https://www.uca.es/wp-content/uploads/2017/01/Código-Ético-UCA.pdf), and that having not studied the subjects, but showing the product of an interaction (meme) of the researcher with her family and social nucleus, we consider that it is not being experimented with human beings.

6 They send the latest memes like the students who always raise their hand first in class.

7 They look at what the others say, but they hardly participate.

8 They access from time to time, without going on sometimes for days or weeks.

9 https://www.youtube.com/watch?v=DpQyuMPrhog

10 https://verne.elpais.com/verne/2020/05/08/articulo/1588930677_213430.html

11 Hugo Molina, 3 years old, was the winner of Spain’s Got Talent (December 2019) for his drumming skills.

12 A portmanteau word is a combination of two words or morphemes, and their definitions, into a new word.

13 Covidiota Covidiot (Covid + idiot) a person who commits irresponsible acts that harm others: ignores social distancing, does not respect rules, does not wear face-masks, etc. “Confitamiento” in allusion to the weight gained, in the absence of physical exercise and given the intake of sweet treats. (Confitar + confinamiento) (Sweets + lockdown). Balconazi (Balcony+nazi): Those who act as balcony cops to insult people on the street because they think they are behaving inappropriately. Cuarenpena (Cuarentena+pena) (Quarantine + sadness): The term refers to the sadness of staying indoors during quarantine to avoid contagion.
