# Supplementary material for: Humor in Times of COVID-19 in Spain: Viewing Coronavirus Through Memes Disseminated via WhatsApp
Source: Front Psychol. 2021 Apr 1;12:611788. doi: 10.3389/fpsyg.2021.611788 (PMC8047868; doi:10.3389/fpsyg.2021.611788)
Supplement: Supplementary file 1 [file Data_Sheet_1.docx]

Humour in times of COVID-19 in Spain: vision of the Coronavirus through Memes disseminated via WhatsApp

Lucía-Pilar Cancelas-Ouviña

Departamento de Didáctica de la Lengua y la Literatura. Faculty of Education. University of Cádiz (Spain) ORCID ID: https://orcid.org/0000-0002-4095-471X

*** Correspondence:**Corresponding Author: Lucía-Pilar Cancelas-Ouviña
[lucia.cancelas@uca.es](mailto:lucia.cancelas@uca.es)

**SUPPLEMENTARY MATERIAL:**

| Text meme^[[1]](#footnote-1)^ | Text+Image Meme^[[2]](#footnote-2)^ | Visual Meme |
| --- | --- | --- |
| 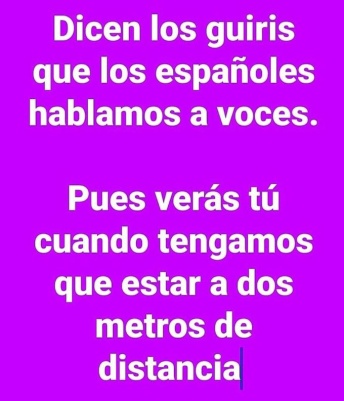 | 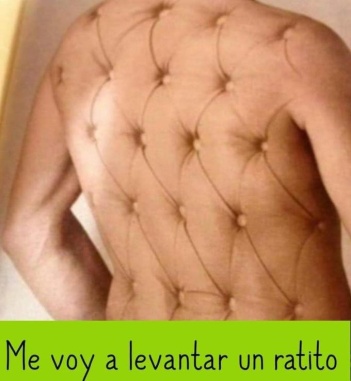 | 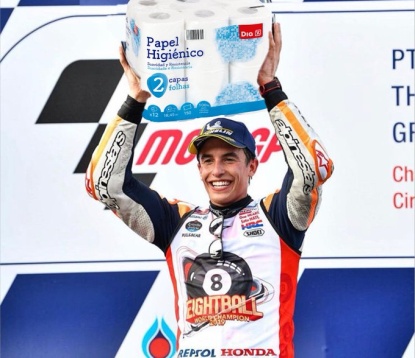 |

Illustration 1: Types of memes according to their design


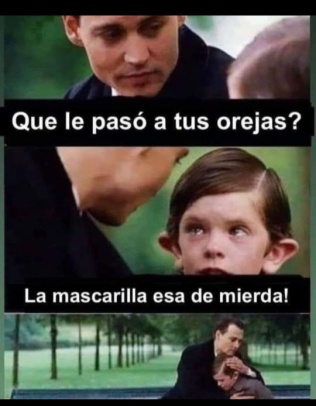


Illustration 2: Example of drakeposting^[[3]](#footnote-3)^

| 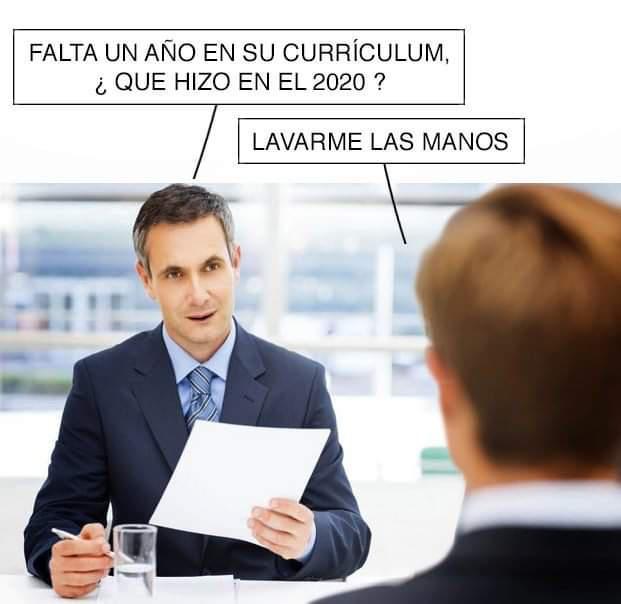 | 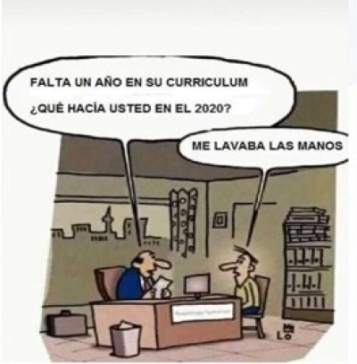 |
| --- | --- |

Illustration 3 ^[[4]](#footnote-4)^


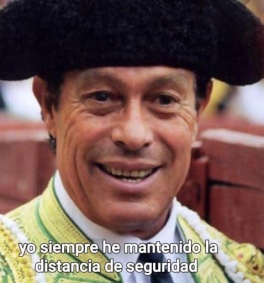


Illustration 4: example of local humour^[[5]](#footnote-5)^

| 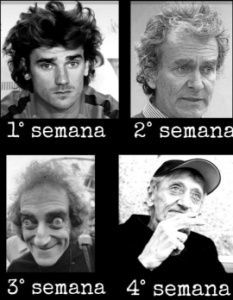 |  |  |  |
| --- | --- | --- | --- |

Illustration 5^[[6]](#footnote-6)^: Example to explain the creative process involved in meme design

| 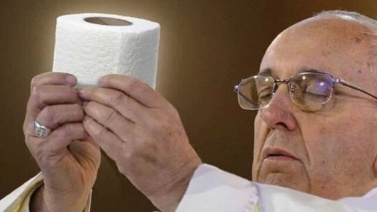 | 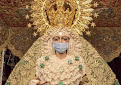 |
| --- | --- |
| Pope Francisco | Virgin Esperanza Macarena (Seville) |

Illustration 6: COVID-19 memes of a religious nature

|  | **Classification categories** | | | **Nº.** | **%** |
| --- | --- | --- | --- | --- | --- |
| 1. | About the COVID-19 virus (Characteristics, effects...) | | | 63 | 9,68% |
| 2. | Main characters in the crisis COVID-19 | | | 75 | 11,52% |
|  | - Fernando Simón (Director of the Health Alert and Emergency Coordination Centre) | 37 | 5,68% |  |  |
|  | - Pedro Sánchez (Prime Minister) | 18 | 2,76% |  |  |
|  | - Comité Expertos COVID-19 | 4 | 0,61% |  |  |
|  | - Salvador Illa (Minister of Health) | 8 | 1,23% |  |  |
|  | - Pablo Iglesias (Deputy Prime Minister) | 6 | 0,92% |  |  |
|  | - Irene Montero (Minister for Equality) | 2 | 0,31% |  |  |
| 3. | Life in Confinement (Stay at Home, isolation, habits, routines...) | | | 40 | 6,41% |
| 4. | COVID-19 Hymn “Resistiré” | | | 4 | 0,61% |
| 5. | Applause and life on the balconies | | | 8 | 1,23% |
| 6. | Disinfection, hand washing and hydroalcoholic gels | | | 13 | 2,00% |
| 7. | Purchasing and supply | | | 56 | 8,60% |
|  | - General comsuption and supply | 15 | 2,30 % |  |  |
|  | - Toilet paper | 41 | 6,30 % |  |  |
| 8. | Food intake and weight gain | | | 31 | 4,76 % |
| 9. | Sedentary lifestyle | | | 5 | 0,77 % |
| 10. | Homeschooling and care of children | | | 24 | 3,69 % |
| 11. | Teleworking | | | 14 | 2,15 % |
| 12. | Dressing and Aesthetics | | | 23 | 3,53 % |
|  | - Clothing and aesthetic care during confinement | 19 | 2,92 % |  |  |
|  | - Hairdresser's shop | 4 | 0,61 % |  |  |
| 13. | COVID-19 and sexual habits | | | 6 | 0,92% |
| 14. | Confinement | | | 32 | 4,92% |
|  | - Prohibition to leave the house | 9 | 1,38 % |  |  |
|  | - Picaresque to skip the norms | 14 | 2,15 % |  |  |
|  | - Walk the dog | 9 | 1,38 % |  |  |
| 15. | On Instructions and measures proposed by the Government | | | 11 | 1,69% |
| 16. | Working conditions of health personnel | | | 15 | 2,30% |
|  | - Lack of medical equipment | 1 | 0,15% |  |  |
|  | - PPE (Personal Protective Equipment) | 6 | 0,92% |  |  |
|  | - Tests COVID-19 | 8 | 1,23% |  |  |
| 17. | Vaccine COVID-19 | | | 5 | 0,77% |
| 18. | Risk groups | | | 3 | 0,46% |
| 19. | Pandemic curve and peak | | | 3 | 0,46% |
| 20. | De-escalation | | | 28 | 4,30% |
| 21. | State of mind during confinement | | | 6 | 0,92% |
| 22. | Desire to go out and boredom | | | 24 | 3,69% |
| 23. | Family life during confinement | | | 9 | 1,38% |
| 24. | Social attitudes and behaviours during the COVID | | | 2 | 0,31% |
| 25. | Things you will do after confinement | | | 18 | 2,76% |
| 26. | Life in the new normal | | | 21 | 3,23% |
| 27. | Masks | | | 48 | 7,37% |
| 28. | Social distance | | | 15 | 2,30% |
| 29. | Suspension of popular festivals | | | 14 | 2,15% |
| 30. | Easter 2020 Holidays | | | 23 | 3,53% |
| 31. | Summer 2020 Holidays | | | 12 | 1,84% |
|  | TOTAL | | | 651 | 100,00% |

Table 1: Memes classified into categories (Own elaboration).

| 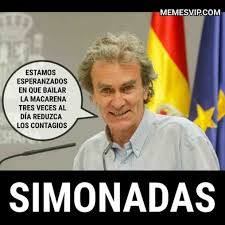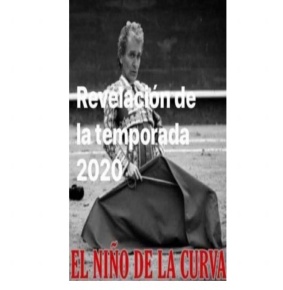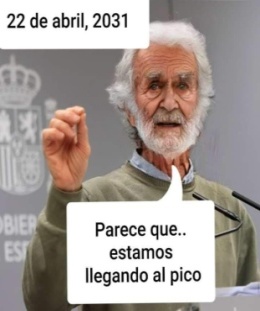 |
| --- |

Illustration 7: Memes on Fernando Simón^[[7]](#footnote-7)^

| 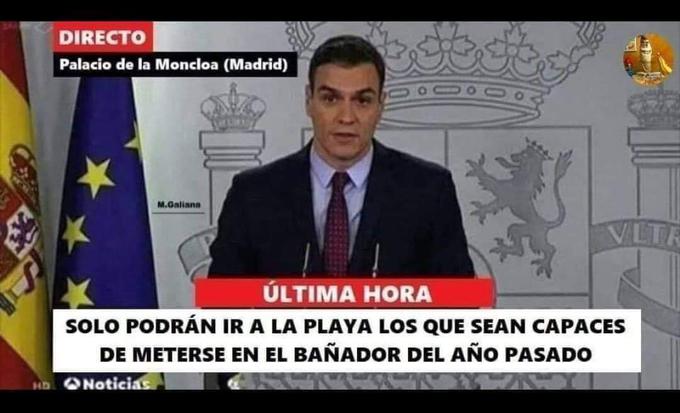 | 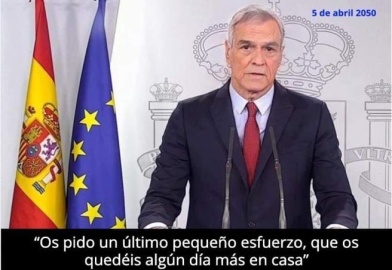 |
| --- | --- |

Illustration 8: Prime Minister Pedro Sánchez^[[8]](#footnote-8)^

| 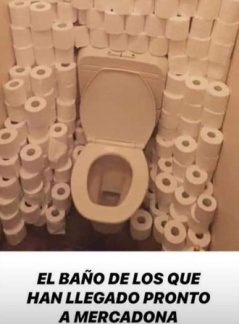 | 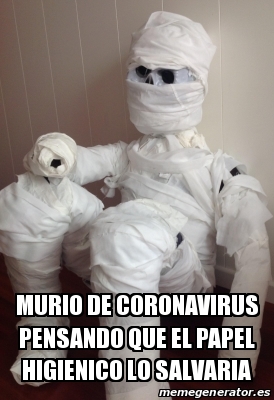 | 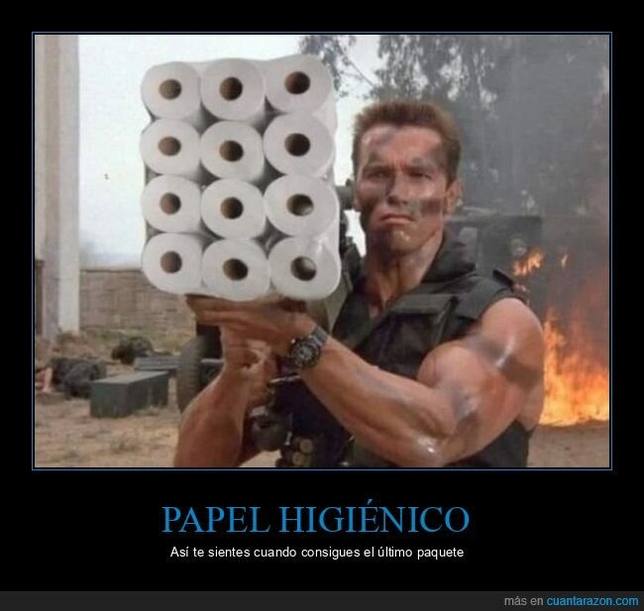 |
| --- | --- | --- |

Illustration 9: Memes on the purchase of toilet paper ^[[9]](#footnote-9)^

| 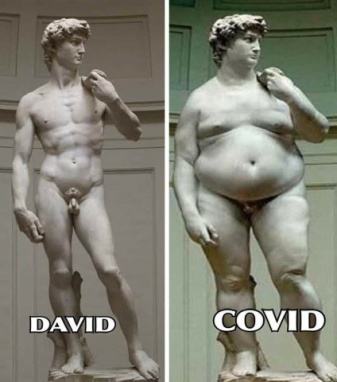 | 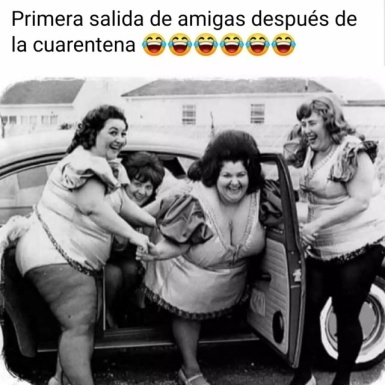 | 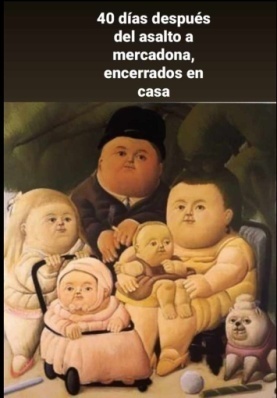 |  |
| --- | --- | --- | --- |

Illustration 10: Memes about overweight during confinement^[[10]](#footnote-10)^

| 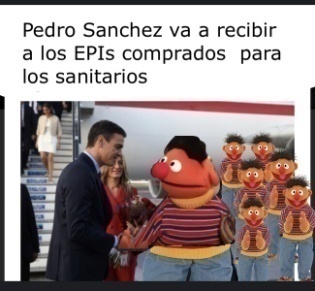 | 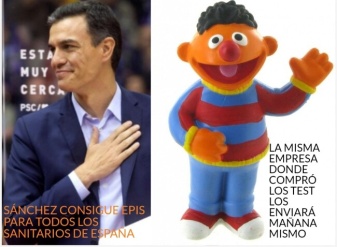 | 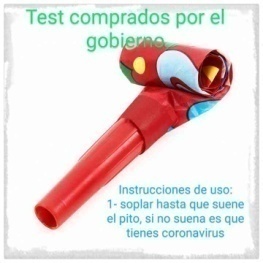 |
| --- | --- | --- |

Illustration 11: Meme on EPIs/PPE and COVID-19 tests^[[11]](#footnote-11)^

| 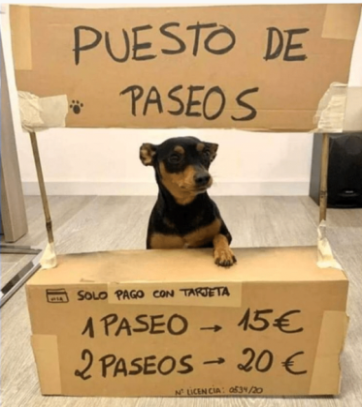 | 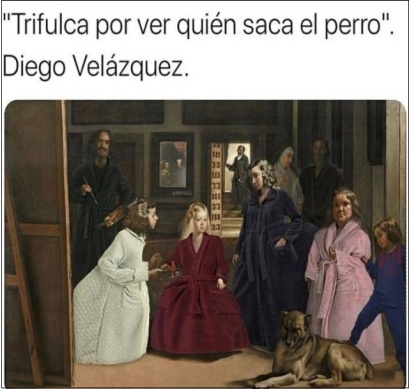 |
| --- | --- |

Illustration 12: Memes on walking the dog^[[12]](#footnote-12)^

| 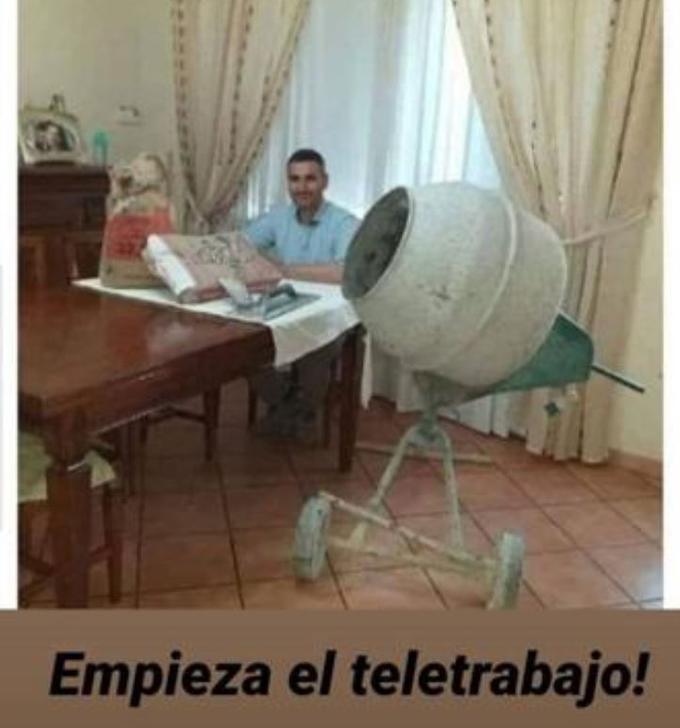 | 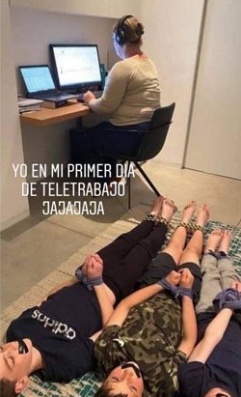 |
| --- | --- |

Illustration 13: Memes on teleworking^[[13]](#footnote-13)^

| \| 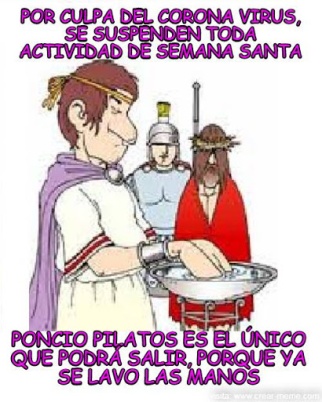 \| \| --- \| | 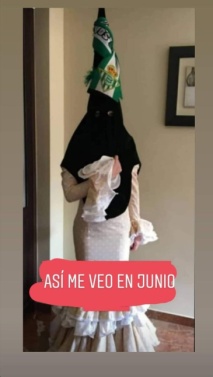 |
| --- | --- | --- |

Illustration 14: Memes on traditional Spanish festivals ^[[14]](#footnote-14)^

| 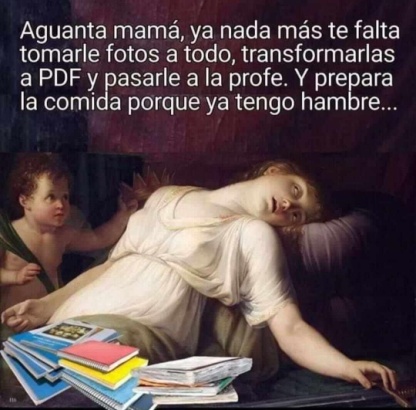 | 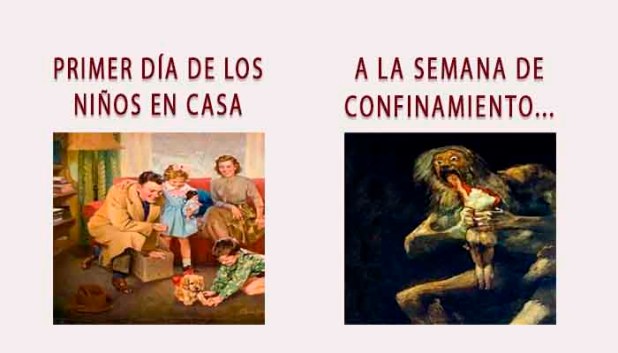 |  |
| --- | --- | --- |

Illustration 15: Memes on homeschooling and childcare ^[[15]](#footnote-15)^

| 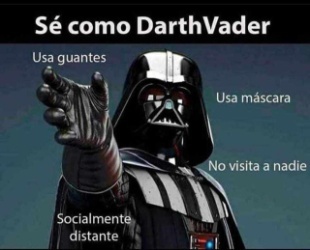 | 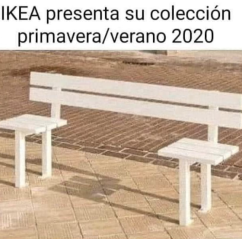 |  |
| --- | --- | --- |

Illustration 16: Memes on the new normal ^[[16]](#footnote-16)^

1. Translation: Guiris (foreigners) say that Spanish people speak very loudly....Well, you´ll see when we have to be two metres apart. [↑](#footnote-ref-1)
2. Translation 1: I'm going to get up for a little while (Alluding to sedentary lifestyles in confinement) [↑](#footnote-ref-2)
3. Translation 2: - What happened to your ears?

   - The stupid mask!! [↑](#footnote-ref-3)
4. Translation: - “There’s a whole year not accounted for in your Curriculum Vitae? What did you do in 2020?”

   – “Washed my hands”. [↑](#footnote-ref-4)
5. Translation: “I have always kept a safe distance”.

   Explanation of the meme: The famous bullfighter Curro Romero is known because he recognized that he was afraid of bulls and he did not get close to bulls. [↑](#footnote-ref-5)
6. Translation 1: Week 1, Week 2, Week 3, Week 4. [↑](#footnote-ref-6)
7. Translation 2: “Simonada”: We hope that dancing the Macarena three times a day will reduce infections.

   Translation 3: Season´s revelation 2020: the child of the curve (Synonym of peak).

   Translation 4: 22nd April 2031: We seem to be reaching the peak. [↑](#footnote-ref-7)
8. Translation1: Breaking News: Only people who can get into last year's swimming costume can go to the beach.

   Translation 2: I ask you to make one last little effort to stay home one more day (5th April 2050). [↑](#footnote-ref-8)
9. Translation 1: The bathroom of those who arrived early at Mercadona (supermarket).

   Translation 2: He died of Coronavirus thinking that toilet paper would save him.

   Translation 3: Toilet paper: that's how you feel when you've got the last pack. [↑](#footnote-ref-9)
10. Translation 1: DAVID – COVID.

    Translation 2: First outing with friends after quarantine.

    Translation 3: 40 days after the assault on Mercadona (supermarket), locked up in the house. [↑](#footnote-ref-10)
11. Translation 1: Pedro Sanchez is going to receive all the “EPIs” purchased for the health personnel.

    Translation 2: Sanchez obtains EPIs for all health personnel. The same company where he bought the tests will send them today.

    Translation 3: Tests bought by the government. Instructions for use: 1. Blow until it makes a noise, if it doesn't, you have Coronavirus. [↑](#footnote-ref-11)
12. Translation 1: Bickering to see who walks the dog. (Las Meninas by Diego Velázquez)

    Translation 2: Walking the dog stand. 1 walk: 15€, 2 walks 20€. Payment by card only. [↑](#footnote-ref-12)
13. Translation 1: Bricklayer says: Working from home begins!!!

    Translation 2:. Me on my first day of working from home, hahaha!. [↑](#footnote-ref-13)
14. Translation 1: Because of Coronavirus, all activity is suspended during Holy Week. Pontius Pilate is the only one who will be able to go out because he has washed his hands.

    Translation 2: This is how I see myself in June (For the accumulation of social events that we expected to have at the end of the year.) [↑](#footnote-ref-14)
15. Translation 1: Hold on mum, you just need to take pictures of everything, upload them in pdfs and send them to the teacher. And make some food because I'm hungry already.

    Translation 2: Children's first day at home...a week into lockdown. [↑](#footnote-ref-15)
16. Translation 1: Be like Darth Vader: wear a mask, wear gloves, do not visit anyone, be socially distant.

    Translation 2: IKEA presents its spring/summer 2020 collection (Allusion to physical distancing). [↑](#footnote-ref-16)
